# Duplex Surface Enhanced Raman Scattering-Based Lateral Flow Immunosensor for the Low-Level Detection of Antibiotic Residues in Milk

**DOI:** 10.3390/molecules25225249

**Published:** 2020-11-11

**Authors:** Ruiqi Fan, Shusheng Tang, Sunlin Luo, Hu Liu, Wanjun Zhang, Chunjiang Yang, Lidong He, Yiqiang Chen

**Affiliations:** 1 State Key Laboratory of Animal Nutrition, College of Animal Science and Technology, China Agricultural University, Beijing 100193, China; fanruiqi94110@cau.edu.cn (R.F.); lsl18810791522@163.com (S.L.); liuhu0674@126.com (H.L.); S20193040574@cau.edu.cn (W.Z.); 2 College of Veterinary Science, China Agricultural University, Beijing 100193, China; tssfj@cau.edu.cn; 3 Ring Biotechnology Co Ltd., Building 7, Bodaxing Industry Park, BDA, Beijing 101111, China; yangchj@nbgen.com; 4 Department of Chemistry and Biochemistry, Florida State University, Tallahassee, FL 32306, USA; cavalierhld@gmail.com

**Keywords:** surface enhanced raman scattering, immunosensor, tetracycline, penicillin, milk

## Abstract

A duplex surface enhanced Raman scattering (SERS)-based lateral flow immunosensor was established for the simultaneous detection of two common antibiotic residues including tetracycline and penicillin in milk. The newly synthesized Au@Ag nanoparticles were labeled with different Raman molecules including 5,5-dithiobis-2-nitrobenzoic acid (DTNB) or 4-mercaptobenzoic acid (MBA), followed by the conjugation of anti-tetracycline monoclonal antibody or anti-penicillin receptor, forming two kinds of SERS nanoprobes. The two nanoprobes can recognize tetracycline-BSA and ampicillin-BSA, respectively, which facilitates the simultaneous detection of the two types of antibiotics on a single test line. After optimization, detection limits of tetracycline and penicillin as low as 0.015 ng/mL and 0.010 ng/mL, respectively, were achieved. These values were far below those of most of other documented bio-analytical approaches. Moreover, the spiking test demonstrates an excellent assay accuracy with recoveries of 88.8% to 111.3%, and satisfactory assay precision with relative standard deviation below 16%. Consequently, the results demonstrate that the SERS-based lateral flow immunosensor developed in this study has the advantages of excellent assay sensitivity and remarkable multiplexing capability, thus it will have great application potential in food safety monitoring.

## 1. Introduction

Antibiotics such as tetracycline and penicillin are frequently used to prevent and treat cow mastitis [1]. However, over-dose use or inadequate withdrawal time can lead to antibiotic residues in milk and cause potential health hazards to consumers [2]. Previous studies indicated that consumption of antibiotic retained milk would pose the risks of allergies, hepatotoxicity, nephrotoxicity, and other direct toxicities to human beings [3]. To prevent the health risk, the authorities have set the maximum residue limits (MRLs) of antibiotic residue in milk [4]. Moreover, antibiotic residue may also lead to bacterial resistance and the predicted-no-effect-concentrations (PNECs) for resistance selection ranging from 8 to 64 pg/mL [5], which is much lower than the regulatory limits. Thus, for ensuring consumer health, it is very necessary to monitor the low-level antibiotic residue in milk.

Currently, the detection of antibiotic residues is mainly conducted by instrumental analysis and immunoassay [6,7,8]. Instrumental analysis typically represented by liquid chromatography-tandem mass spectrometry (LC-MS/MS) shows high sensitivity, high assay accuracy, and precision [7], but requires prohibitive instruments, well-trained personnel, and generally complex sample pretreatment, which limited their application in the field environment [8]. Because rapid and cost-effective tests of antibiotic residues are required in the dairy industry, diverse immunoassays have been widely used for this purpose [6].

Among the immunoassays, lateral flow immunoassay (LFIA) is the most often used method for antibiotic residue detection, with the advantages of rapidity, simplicity, and cost-effectiveness [9]. Nevertheless, traditional lateral flow immunoassay generally employs gold nanoparticle (AuNPs) as labels for colorimetric detection and suffers from poor assay sensitivity and limited multiplexing capability. By comparison, surface enhanced Raman scattering (SERS) nanotag is proved as an excellent label of immunoassay [10]. The SERS enhancement effect originates from the combination of electromagnetic and chemical enhancement when the Raman labeling molecules are adsorbed onto the surface of gold or silver nanoparticle [11]. It has several advantages including the strong molecular characteristics of Raman labels, single wavelength excitation for multiple Raman labels, and the resistance to self-quenching [12]. Thus, SERS-based immunosensors have been widely used for biomedicine diagnosis and food safety monitoring [11,12,13,14,15]. In recent years, by combining the lateral flow assay format and SERS-based immunosensor, many SERS-based immunosensing methods have been established for the detection of various target analytes, e.g., respiratory viruses [16], human chorionic gonadotropin [17], and interleukin-6 [18]. Furthermore, some multiplex SERS-based immunosensors have also been developed for the detection of cardiac biomarkers [19], neomycin and quinolone antibiotics [20], nucleic acids [21], *L. monocytogenes*, and *S. Enteritidis* [22], by establishing two or three test lines on different positions of nitrocellulose membrane. However, the multiplexing capability of this format is still limited because the detection zone of nitrocellulose membrane is restricted and cannot accommodate too many test lines. In this study, we utilized two Raman reporters with distinct spectra for labeling the newly synthesized Au@Ag core–shell nanoparticle (Au@Ag NP) and developed a duplex SERS-based immunosensor for the simultaneous detection of two types of antibiotic residues on a single test line, which can greatly extend the multiplexing detection capability of lateral flow immunoassay. Moreover, we developed and employed a high-affinity anti-tetracycline monoclonal antibody and anti-penicillin receptor for the preparation of SERS nanoprobes, which facilitates the ultra-sensitive detection of the antibiotic residues in milk.

## 2. Results and Discussion

### 2.1. Scheme of Duplex SERS-Based Lateral Flow Immunosensor

As indicated in Figure 5, the nitrocellulose membrane was immobilized with two capture antigens including tetracycline-BSA and ampicillin-BSA conjugates, while the micro-plate well contained two kinds of SERS nanoprobes including anti-tetracycline Au@Ag NP-DTNB-antibody conjugate and anti-penicillin Au@Ag NP-MBA-receptor conjugate. If the sample solution did not contain tetracycline, penicillin, and related analogs, the SERS nanoprobes could bind with the capture antigens on the test line to form a visible test line. Beneath the laser excitation, an intensive SERS signal (1332 cm^−1^ for tetracycline and 1079 cm^−1^ for penicillin) can be produced (Figure 5). Nevertheless, if the sample solution contained tetracycline or penicillin molecules, the antibiotics in the solution can first bind with the SERS nanoprobe in the micro-plate well, and less free SERS nanoprobes would react with the capture antigen on the nitrocellulose membrane, and hence less SERS nanoprobes would be captured by the coating antigen. Consequently, a far weaker SERS signal would be produced on the test line of the nitrocellulose membrane. If tetracycline and penicillin were in an excess amount, the detection reagents would be impeded from mixing with the capture reagents, and no corresponding SERS signal on the test line can be produced (Figure 5). If the SERS-based LFIA process was normal, a visible control line could be observed by the naked eye and intensive SERS signals can be produce under lasers’ excitation. For the quantitative determination of antibiotic residue, two calibration curves were constructed by plotting the ratios (B/B_0_) of SERS intensity between the spiked and blank samples against the logarithmic concentrations of corresponding antibiotics. The analyte concentration can be determined by the collected signal of an unknown sample based on the established calibration curve.

### 2.2. SERS Nanoprobe Preparation

**Figure 1:**
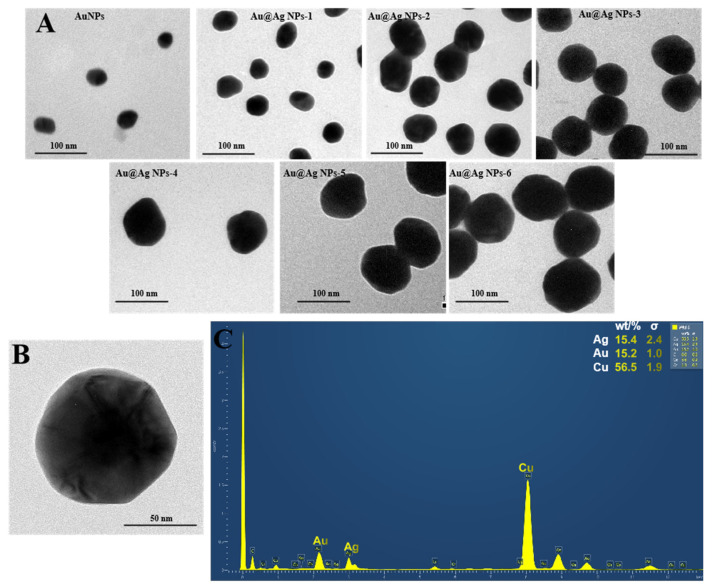
The transmission electron microscopy (TEM) images (**A**) of the synthesized AuNPs and Au@Ag NPs, the field emission (FETEM) image (**B**) of typical Au@Ag NP, and the element mapping image (**C**) of energy-dispersive X-ray spectroscopy (EDS) on Au@Ag NP. The sizes of the AuNPs and the six Au@AgNPs were measured by TEM to be 32.2 ± 3.3, 41.8 ± 3.9, 50.3 ± 5.8, 62.5 ± 7.4, 76.9 ± 8.7, 92.2 ± 10.8, and 99.6 ± 13.1 nm (*n* = 30), respectively.

As previous studies have demonstrated that a silver surface can produce stronger SERS enhancement than that of gold substrates [23], Au@Ag NPs were prepared and used for the preparation of SERS nanoprobes. Moreover, different sizes of Au@Ag NPs were synthesized by altering the added volume of silver-staining solution. As shown in Figure 1, the sizes of these Au@Ag NPs were gradually increased from 32 to 98 nm (Figure 1A), as indicated by the TEM images. To further characterize the core–shell structure of Au@Ag NP, an FETEM scanning was performed and the images showed that the synthesized nanoparticle contained an obvious core–shell structure (Figure 1B). The energy-dispersive X-ray spectroscopy (EDS) analysis was also performed and the result (Figure 1C) showed that the Au@Ag NP contains both Au and Ag elements. All of these results demonstrate that the Au@Ag NPs were well prepared and can be used in the following experiment.

**Figure 2:**
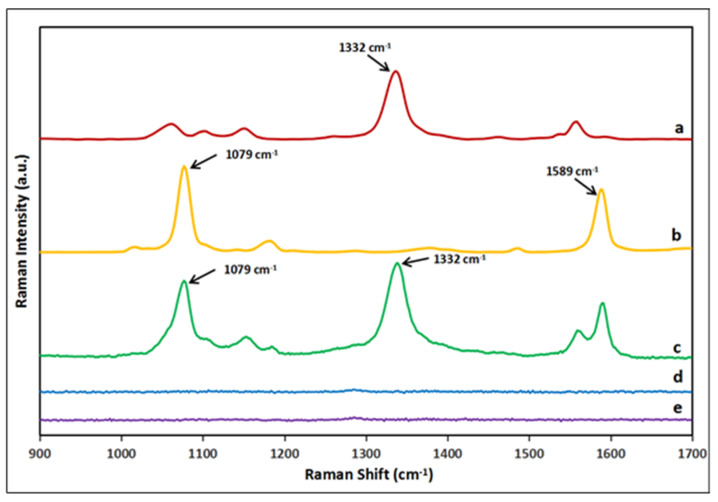
The Raman spectra of DTNB and MBA on Au@Ag NP. a–e represent the following: (a) the Raman spectra of Au@Ag NP-DTNB; (b) Au@Ag NP-MBA; (c) the mixture of Au@Ag NP-DTNB and Au@Ag NP-MBA; (d) the mixture of free DTNB and MBA; and (e) Au@Ag NPs.

SERS nanoprobe was prepared by modifying Au@Ag NP with Raman labels and antibody/receptor. Firstly, a layer of DTNB or MBA molecules was placed on the Au@Ag NP via Au-S bond. Both DTNB and MBA have been utilized as Raman labels owing to their inherent capacity to generate a characteristic and intense SERS signal [10,12]. Figure 2 shows the characteristic peaks of DTNB mainly comprise 1059, 1332, and 1558 cm^−1^, while the typical peaks of MBA mainly comprise 1079 and 1589 cm^−1^, respectively. As the bands of 1332 cm^−1^ of DTNB and 1079 cm^−1^ of MBA are the most intense bands in their spectra, the SERS intensity of two bands was thus used for quantitative analysis. It can be observed that the two bands from DTNB and MBA have no significant overlap with each other, thus facilitating the simultaneous detection of two different antibiotic residues on a single test line by utilizing DTNA and MBA as Raman labels in the preparation of SERS nanoprobes. To further confirm the SERS enhancement effect originated from Au@Ag NPs, the Raman signal of both Au@Ag NP-DTNB/MBA complexes and free DTNB/MBA molecules was compared. As revealed in Figure 2, the Au@Ag NP-DTNB/MBA complexes produced an intensive Raman signal at 1332 cm^−1^ and 1079 cm^−1^, respectively. Nevertheless, free DTNB and MBA molecules at the same Raman reporter concentration did not produce any observable Raman signal. Furthermore, the Au@Ag NPs cannot yield any observable Raman signal (Figure 2a). Thus, it can be concluded that the Raman signals of DTNB and MBA were greatly enhanced by the Au@Ag NPs after the two Raman labels were attached onto the Au@Ag NP surface.

**Figure 3:**
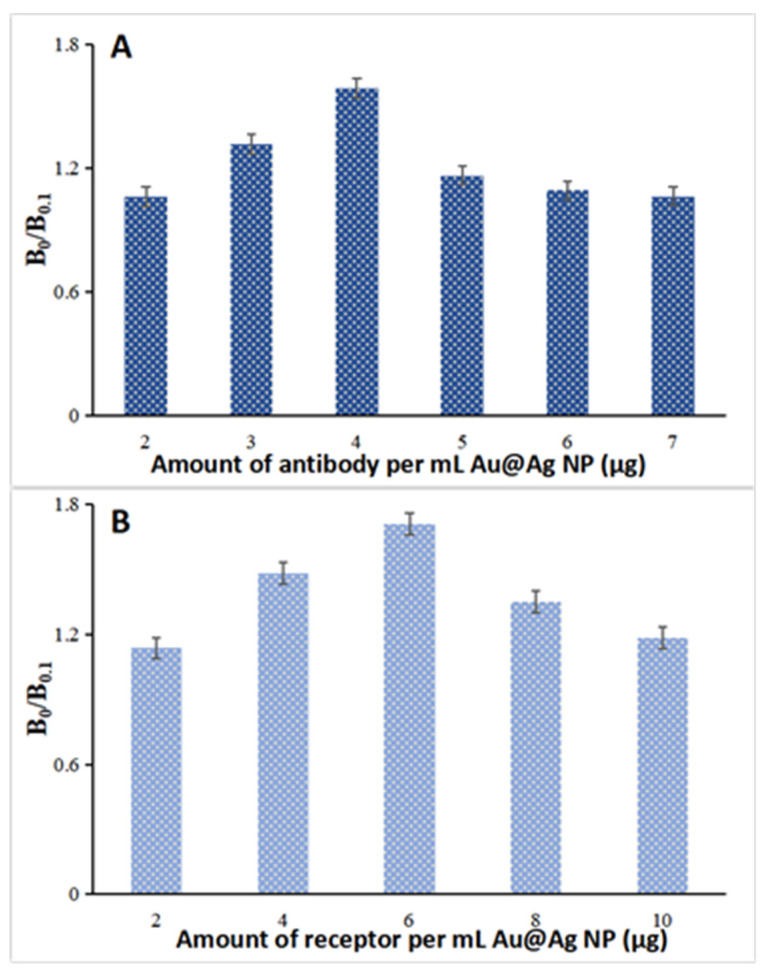
The effects of anti-tetracycline antibody amount (**A**) and anti-penicillin receptor amount (**B**) on SERS-based lateral flow immunoassay sensitivities.

After attaching DTNB or MBA molecules onto the Au@Ag NP surface, the antibody/receptor could be passively adsorbed onto the nanoparticle surface via electrostatic and hydrophobic interaction [24]. The antibody/receptor amount used in the conjugation process is anticipated to affect the attached antibody/receptor number per Au@Ag NP. This is correlated to the SERS nanoprobe avidity and influences the assay sensitivity [24]. To optimize the antibody/receptor amount for SERS nanoprobe preparation, the ratio of SERS intensity at 0 and 0.1 ng/mL of tetracycline or penicillin (B_0_/B_0.1_) was compared for the prepared nanoprobes. Consequently, at a similar SERS signal, the optimized amounts of antibody/receptor that generate the most significant difference between 0 and 0.1 ng/mL of the antibiotics were measured to be 4.0 μg of anti-tetracycline antibody (Figure 3A) and 6.0 μg of anti-penicillin receptor (Figure 3B). Thus, these antibody/receptor amounts were chosen for preparing the SERS nanoprobes.

### 2.3. SERS-Based Lateral Flow Immunosensor

**Figure 4:**
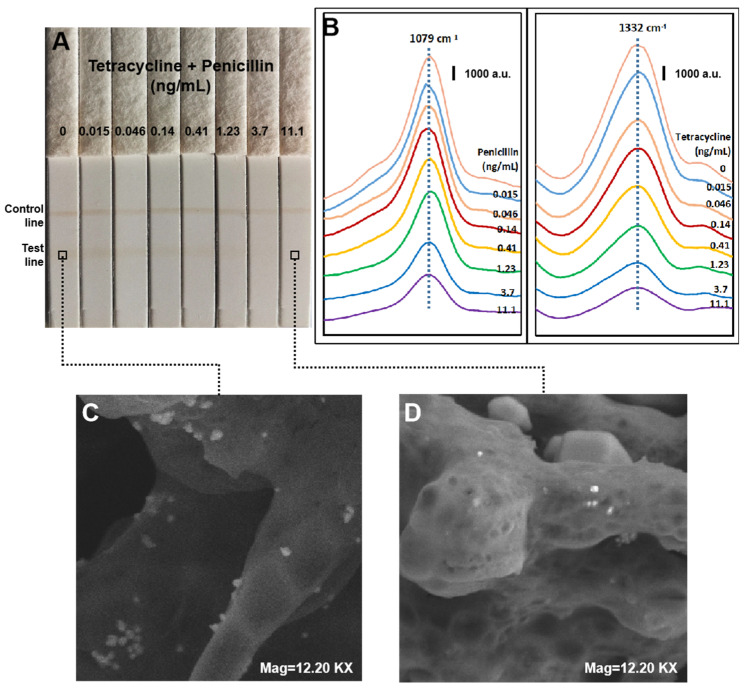
Typical photo images of SERS-based lateral flow strips (**A**), SERS spectra (**B**), and scanning electron microscope (SEM) images (**C**,**D**).

As tetracycline and penicillin are both small molecules, the SERS-based lateral flow immunosensor utilized a competitive assay format. In this format, free tetracycline/penicillin molecules and capture antigens compete for the limited antibody/receptor epitopes on the SERS nanoprobes. The analyte concentrations in samples can be determined through the Raman signal produced by the SERS nanoprobes captured on the test line of lateral flow strips (Figure 4). Figure 4A,B show typical photographic images obtained from the test strips and their corresponding SERS spectra are depicted. Increasing the concentration of tetracycline and penicillin decreased all the characteristic peaks of SERS spectra, which is typically observed for a competitive immunoassay. Moreover, as shown in the SEM images of the lateral flow strip, for the negative sample, many SERS nanoprobes were bound on the test line (Figure 4C), while for the positive sample, less SERS nanoprobes were observed to be attached onto the test line (Figure 4D). This further confirms that the characteristic Raman signals were produced by the SERS nanoprobes. The calibration curves as indicated in Figure 4B plot the Raman signal at 1332 cm^−1^ and 1079 cm^−1^, the most characteristics peaks of DTNB and MBA, against the logarithmic concentrations of tetracycline or penicillin. As evident from the figure, the SERS intensities demonstrate a linear regression with a logarithmic rise in analyte concentration and the linear ranges were in the range of 0.02–11 ng/mL for both tetracycline and penicillin in milk.

Next, we examined the detection specificity of the duplex SERS-based immunosensor. As indicated in Figure S1, strong and characteristic SERS signals on test line were obtained when the test procedure was correctly conducted and the anti-tetracycline and anti-penicillin SERS nanoprobe separately recognized the tetracycline-BSA and ampicillin-BSA conjugate. However, under laser excitation, no SERS signal was achieved if the coating antigens tetracycline-BSA and ampicillin-BSA were replaced with BSA. Likewise, no SERS signal was detected if the anti-tetracycline mAb and anti-penicillin receptors on the SERS nanoprobes were replaced with other mAbs. These results suggest that the newly designed anti-tetracycline and anti-penicillin SERS nanoprobes were very specific to the tetracycline-BSA and ampicillin-BSA conjugates immobilized on the test line.

### 2.4. Method Validation

The established SERS-based lateral flow immunosensor was validated by a spiked recovery experiment (The curves were shown in Figure S2) according to the Analytical Procedures and Methods Validation for Drugs and Biologics established by the Food and Drug Administration [25]. Blank milk samples were verified by LC-MS/MS analysis to be free of tetracycline and β-lactam antibiotics. This blank sample was then spiked with serial diluted standard solutions of tetracycline and penicillin as reference analytes. The spiked samples were then analyzed by the developed immunosensor. The limits of detection (LODs) were clarified as the concentrations of tetracycline or penicillin resulting in a 10% decrease in the respective SERS signal related to the blank sample [26]. By calculation, the LODs were determined as 0.015 ng/mL of tetracycline and 0.010 ng/mL of penicillin in milk. These values are much lower than the maximum residue limits (100 ng/mL for tetracycline and 4 ng/mL for penicillin) established by the authorities [4]. By comparison, the assay sensitivities of the SERS-based lateral flow immunosensor are higher than those of other LFIAs [8], and are also better than those of most other immunoassays [2,8,26,27,28,29,30,31,32,33] reported in the literature (Table 1). Furthermore, the duplex SERS-based immunosensor can simultaneously detect two different types of chemicals on a single test line, which could greatly extend the multiplexing capability of the SERS-based immunosensor. Additionally, as the used anti-tetracycline antibody can recognize 4 tetracycline antibiotics and the anti-penicillin receptor can recognize 13 β-lactam antibiotics (Table S1), this developed duplex SERS-based immunosensor has the potential to simultaneously detect all 4 tetracyclines and 13 β-lactam antibiotics, respectively. In order to evaluate the specificity of this dual SERS-based immunosensor, high concentrations of common antibiotics used in dairy cows including streptomycin, neomycin, lincomycin, erythromycin, and chloramphenicol were separately spiked into blank milk samples and then detected by the immunosensor. It was found that these antibiotics would not interfere with the detection of both tetracycline and penicillin by the immunosensor.

The accuracy and precision of this SERS-based lateral flow immunosensor were then assessed. The results showed that the spiked recoveries of both antibiotics at 2 to 10 ng/mL ranged from 88.8% to 111.3%, and the relative standard deviations (RSDs) were below 16% (Table 2). This result indicated that the assay accuracy and precision of this method were satisfactory. Furthermore, the reproducibility of this SERS-based immunosensor was evaluated by determining 0.5 and 5.0 ng/mL of tetracycline and penicillin spiked in blank milk sample using different batches of lateral flow strips and SERS nanoprobes. The result showed that the variations for all of the analytical values were lower than 18% (Table S2). This implied that the preparations of lateral flow strips and the SERS nanoprobe were both reproducible. Therefore, the constructed duplex SERS-based immunosensor can be utilized for the rapid analysis of tetracycline and penicillin in milk.

## 3. Materials and Methods 

### 3.1. Materials

Chloroauric acid, 5,5’-dithiobis-2-nitrobenzoic acid (DTNB), 4-mercaptobenzoic acid (MBA), bovine serum albumin (BSA), and Tween 20 were bought from Sigma Aldrich (St. Louis, MO, USA). Tetracycline, penicillin, and other chemical standards were bought from China Institute of Veterinary Drug Control (Beijing, China). Common chemical reagents were provided by Beijing Regent Corporation (Beijing, China). Nitrocellulose membrane CN 140 was purchased from Whatman International Ltd (Middlesex, UK). Sample pad (GF2-II) and absorbent pad were bought from Jieyi Biotechnology Co., Ltd. (Shanghai, China). Tetracycline-BSA and ampicillin-BSA conjugates, anti-tetracycline monoclonal antibody (mAb), and anti-penicillin receptor were produced in our research group (Supporting Information). The strip assembly was performed on Biodot XYZ 3050 Dispensing Platform and CM4000 Cutting Module (Biodot Inc., Irvine, CA, USA). The ultra-pure water was prepared by Milli-Q water purification system (Millipore Inc., St. Louis, MO, USA).

### 3.2. Au@Ag NPs’ Synthesis 

Firstly, gold nanoparticles (AuNPs) were synthesized according to the procedure of Frens [34], with a small modification (Supporting Information). Briefly, 100 mL of ultra-pure water was heated to boiling point, and then 1 mL of 1% (*w*/*v*) chloroauric acid aqueous solution was added. The mixed solution was continuously heated and then 1.2 mL of tri-sodium citrate (1.0%, *w*/*v*) was supplemented with constant stirring. The reaction was continued for 20 min and the resulting AuNP solution was allowed to cool at room temperature. Lastly, the volume of the AuNP solution was supplemented to 100 mL. Subsequently, the Au@Ag core–shell NPs were prepared [24]. Briefly, 10 mL of AuNPs (32 nm) synthesized as above was sequentially added with ascorbic acid (60 μL, 0.2 M) and silver nitrate (15 μL, 0.2 M). Then, the reaction was continued for 30 min while the mixture was slowly stirred. Subsequently, another aliquot of ascorbic acid (60 μL, 0.2 M) and silver nitrate (15 μL, 0.2 M) was added for the second cycle. For the synthesis of Au@Ag NPs with different size, different cycles of reaction were performed and the resulting nanoparticles were centrifuged for 20 min and re-dispersed in 10 mL water. A small aliquot of the particle solution was loaded and dried on Cu plate and the sizes of nanoparticles were measured by transmission electron microscopy (TEM, JEOL Inc., USA) and analyzed by DigitalMicrograph 3.5 (Gatan Inc., Pleasanton, CA, USA). The core–shell structure of the Au@Ag NPs was characterized by field emission transmission electron microscopy (FETEM, Tecnai G2-F20, FEI, Hillsboro, OR, USA).

### 3.3. SERS Nanoprobe Preparation 

Briefly, 10 mL of the prepared Au@Ag core–shell NPs (92.2 nm or 50.3 nm) was added with 500 μL of borate buffer (200 mM, pH 8.5). Then, it was mixed with 300 μL of DTNB (1 mM, dissolved in acetonitrile) or 100 μL of MBA (1 mM, dissolved in acetonitrile), respectively. The reaction was left for 30 min at room temperature followed by separate addition of 40 μL anti-tetracycline mAb (1 mg/mL) or 60 μL anti-penicillin receptor (1 mg/mL). The solutions were slightly mixed and incubated for 1 h. Lastly, the Au@Ag NP-DTNB-antibody and Au@Ag NP-MBA-receptor conjugates were mixed with 1 mL of BSA aqueous solution (1%, *m*/*v*, dissolved in 2 mM borate buffer) and the uncoated sites on the surface of the Au@Ag NP surface were blocked. To remove any unreacted reagents, the solutions were centrifuged at 2000 rpm (for 92.2 nm Au@Ag NP) or 3000 rpm (for 50.3 nm Au@Ag NP) for 10 min. The precipitate was then re-suspended with 10 mL of phosphate buffer (10 mM, pH 7.4) containing 0.1% BSA and 0.5% Triton X-100. Finally, 40 μL of each SERS nanoprobe was pipetted into each micro-plate well followed by freeze-drying of the liquid.

### 3.4. Lateral Flow Strip Assembly 

**Figure 5:**
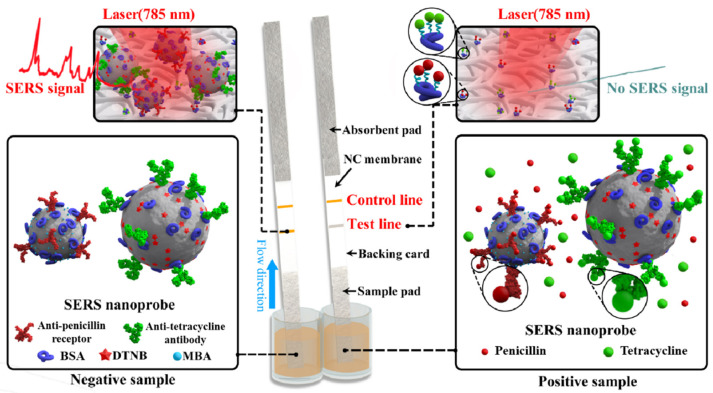
Illustration of surface enhanced Raman scattering (SERS)-based lateral flow immunosensor for tetracycline and penicillin in milk. NC, nitrocellulose; DTNB, 5,5’-dithiobis-2-nitrobenzoic acid; MBA, 4-mercaptobenzoic acid; BSA, bovine serum albumin.

Tetracycline-BSA and ampicillin-BSA conjugates were firstly equally mixed and then dispensed on a nitrocellulose membrane as the test line. Then, the secondary goat anti-mouse antibody (0.6 mg/mL) was dispensed on the nitrocellulose membrane as the control line (Figure 5). The applied volumes of both antigens and second antibody were both 1 μL for the one centimeter line. The nitrocellulose membrane was then allowed dry by being kept at 37 °C for 6 h. The assembly procedure of the lateral flow strip (Figure 5) was as follows. The nitrocellulose membrane previously coated with capture antigens and second antibody was fixed on the center of backing card. Then, the sample pad (woven meshes material) used for loading sample solution was fixed on one side and partly (2 mm) concealed the nitrocellulose membrane. The absorbent pad (paper material) used for absorbing sample solution was fixed on the opposite side of the backing card and also partly (2 mm) concealed the nitrocellulose membrane. Lastly, for each strip, the card was sliced into strips and the width of each strip was 4 mm.

### 3.5. SERS-Based Immunosensing Assay

The duplex SERS-based immunosensing assay was performed in a micro-well format (Figure 5). In brief, 200 μL of milk sample solution was added in a micro-plate well, which contained the detection reagent. The sample solution was then thoroughly mixed with the detection reagent and the solution was incubated at room temperature for 3 min. Then, the sample pad side of a lateral flow strip was put into the micro-plate well. The solution in the well then migrated towards the absorbent pad of the strip. After 8 min, the reaction was then terminated by taking the strip out from the micro-well. The SERS spectra of nanoprobes captured on the test line and control line were then acquired using portable i-Raman^®^ Plus System. This Raman system was equipped with an excitation laser (785 nm), a video microscope sampling system, and a charge-coupled device detector (B&W TEK, Newark, NJ, USA). The Raman spectra were collected from 175 to 3200 cm^−1^ and the spectral resolution was 4.5 cm^−1^. The laser power was set at 50 mW and the integration time was 2000 ms for each point. All the Raman signals were treated by BWSpec Operation Software.

### 3.6. Spiked Recovery Experiment 

The SERS-based lateral flow immunosensor was checked for accuracy and precision by a spiked-recovery experiment. Briefly, blank milk samples (total of 12 samples) were spiked with tetracycline and penicillin standard solutions to produce serial diluted antibiotic concentration. Then, the spiked milk samples were centrifuged at 6000 g for 6 min. Following this, 1 mL of the supernatant was equally mixed with PBS (Phosphate Buffer Saline) containing 1% Triton and 1% BSA. The mixed solution (200 μL) was then added into the micro-plate well and applied to the duplex SERS-based LFIA.

## 4. Conclusions

In this study, a SERS-based lateral flow immunosensor was developed for the low-level detection of tetracycline and penicillin in milk. The utilization of dual Raman labels facilitated the simultaneous detection of the two antibiotics on a single test line of the lateral flow strip. Moreover, the optimization of SERS nanoprobe preparation and the use of high-affinity receptor/antibody both contributed to the ultra-sensitive detection of the two antibiotic residues. Conclusively, the developed immunosensor has excellent assay sensitivity, satisfactory assay accuracy and precision, as well as remarkable multiplexing detection capability. With the development of a low-cost and user-friendly Raman spectrometer, the immunosensor will show great promises for further application in the field of food safety monitoring.

## Figures and Tables

**Table 1:** Brief literature reviews about immunoassays of tetracycline and penicillin B in milk. SERS, surface enhanced Raman scattering; LOD, limit of detection.

Method	Analyte	LOD	Assay Time	Reference
Enzyme immunoassay	Penicillin G	0.1 ng/mL	>60 min	[26]
Fluorometric lateral flow immunoassay	Tetracycline	20 ng/mL	>10 min	[27]
Multiplex lateral flow immunoassay	Penicillin G	0.05 ng/mL	～20 min	[28]
	Tetracycline	0.04 ng/mL		
Immunochromatographic assay	Penicillin G	0.5 ng/mL	～20 min	[29]
Microfluidic immunoassay	Tetracycline	1.01 μg/kg	～17 min	[30]
Quantum dot-based fluorescence immunoassay	Penicillin G	0.005 ng/mL	～90 min	[31]
	Tetracycline	0.005 ng/mL		
Colorimetric immunosensor	Penicillin G	1 ng/mL	5 min	[32]
Potassium thiocyanate (KSCN)-mediated Fe-T1 sensor	Tetracycline	2.31 ng/mL	>2 h	[33]
SERS-based immunosensor	Tetracycline Penicillin G	0.015 ng/mL 0.010 ng/mL	～20 min	This study

**Table 2:** Spiked recoveries and relative standard deviations (RSDs) of tetracycline and penicillin Figure 3.

Analytes	Spiked Level (ng/mL)	Detected Level (Mean Value ± SD, ng/mL)	Recovery (%)	RSD (%)
Tetracycline	2.0	2.14 ± 0.32	106.9	15.8
	4.0	3.55 ± 0.54	88.8	13.6
	10.0	9.63 ± 1.18	96.3	11.8
Penicillin	2.0	2.15 ± 0.20	107.5	10.2
	4.0	4.45 ± 0.44	111.3	10.9
	10.0	9.07 ± 0.96	90.7	9.6

## Supplementary Materials

Figure S1: The specificity of the SERS-based lateral flow immunosensor for tetracycline and penicillin. Figure S2. The calibration curves of tetracycline (A) and penicillin (B) in milk by SERS-based lateral flow immunosensor. Table S1. The IC_50_ and cross-reactivity values of anti-tetracycline mAb and anti-penicillin receptor. Table S2. The reproducibility of SERS-based lateral flow immunosensor for tetracycline and penicillin in milk.
