# Supplementary material for: Duplex Surface Enhanced Raman Scattering-Based Lateral Flow Immunosensor for the Low-Level Detection of Antibiotic Residues in Milk
Source: Molecules. 2020 Nov 11;25(22):5249. doi: 10.3390/molecules25225249 (PMC7698115; doi:10.3390/molecules25225249)
Supplement: Supplementary file 1 [file molecules-25-05249-s001.pdf]

Supplementary Information

Duplex Surface Enhanced Raman Scattering-based Lateral Flow Immunosensor for the Low-level Detection of Antibiotic Residues in Milk

Ruiqi Fan [1,†], Shusheng Tang [2,†], Sunlin Luo [1], Hu Liu [1], Wanjun Zhang [1], Chunjiang Yang [3], Lidong He [4] and Yiqiang Chen [1,*]

[1] State Key Laboratory of Animal Nutrition, College of Animal Science and Technology, China Agricultural University, Beijing 100193, China; fanruiqi94110@cau.edu.cn (R.F.); lsl18810791522@163.com (S.L.); liuhu0674@126.com (H.L.); S20193040574@cau.edu.cn (W.Z.)

[2] College of Veterinary Science, China Agricultural University, Beijing 100193, China; tssfj@cau.edu.cn

[3] Ring Biotechnology Co Ltd, Building 7, Bodaxing Industry Park, BDA, Beijing 101111, China; yangchj@nbgen.com

[4] Department of Chemistry and Biochemistry, Florida State University, Tallahassee, FL 32306, USA; cavalierhld@gmail.com

* Correspondence: yqchen@cau.edu.cn; Tel.: +86-010-6273-3666
† These authors contributed equally to this work.

Received: 25 September 2020; Accepted: 2 November 2020; Published: date

Received: date; Accepted: date; Published: date

List of contents:

1. Preparation of tetracycline-BSA and ampicillin-BSA conjugates.
2. Preparation of monoclonal antibody against tetracycline.
3. Preparation of anti-penicillin receptor
4. Fig. S1. The specificity of the SERS-based lateral flow immunosensor for tetracycline and penicillin.
5. Figure S2. The calibration curves of tetracycline (A) and penicillin (B) in milk by SERS-based lateral flow immunosensor.
6. Table S1. The IC_{50} and cross-reactivity values of anti-tetracycline mAb and anti- penicillin receptor.
7. Table S2. The reproducibility of SERS-based lateral flow immunosensor for tetracycline and penicillin in milk.

1. Preparation of tetracycline-BSA and ampicillin-BSA conjugates

Tetracycline-BSA was synthesized according to the protocol of Zhang et al. [1]. Briefly, 20 mg of tolidine was dissolved in 4 mL of 0.2 N HCl and 20 mg of sodium nitrite was dissolved in 0.5 mL of distilled water. With constant stirring, the sodium nitrite solution was drop-wise added into the tolidine solution at 4 oC and in a dark environment. The mixture was allowed to react for 45 min. Afterwards, 1.0 mL of the mixture was drop-wise added to 3.0 mL of 0.1 M sodium borate solution (pH 8.0) containing 10 mg of BSA and 10 mg of tetracycline. The mixture was allowed to react for 2 h at 4 oC and in a dark environment. Finally, the product was dialyzed against PBS for 24 h to remove unreacted small chemicals.

Ampicillin-BSA was synthesized according to the protocol of Bacigalupo et al. [2]. Briefly, 10 mg of ampicillin and 10 mg of BSA were dissolved in 50 mL of 0.1 M phosphate buffer (PB, pH6.5). Then 1.0 mL of freshly prepared 1% glutaraldehyde solution was added drop-wise. After the reaction mixture was gently stirred for 20 min, sodium borohydride was added to a final concentration of 10 mg/mL, and the solution was incubated for 1 h at 4 °C. Finally, the reaction product was dialyzed against phosphate buffer saline (PBS, 0.01 M, pH 7.4) for 24 h to remove unreacted small chemicals.

2. Preparation of monoclonal antibody against tetracycline

The procedures for the production of monoclonal antibody were based on the commonly used method in our lab. Ten BALB/c female mice (8 weeks old, ten for each conjugate) were immunized with tetracycline-BSA. For the first immunization, 100 μg of immunogen dissolved in PBS (0.01 M with 0.15 M sodium chloride, pH 7.4) was mixed with equal Freund's complete adjuvant and then injected subcutaneously. Two subsequent injections were given at two-week intervals with the same dosage of immunogen emulsified in Freund's incomplete adjuvant. Antisera were collected one week after the third immunization and were screened for their recognition activities against respective mycotoxin by competitive inhibition ELISA (ci-ELISA). The mouse showing the highest reactivity against tetracycline received a fourth injection intraperitoneally (i.p). Four days later, the mouse spleen was removed for hybridoma production. Cell fusion procedure was carried out according to the procedures as described by our group. Briefly, mouse spleen lymphocytes were fused with myeloma cells at a 5:1 ratio using Hybri-Max™ PEG 1450 as the fusing agent. The fused cells were suspended in HAT-RPMI 1640 medium (supplemented with 10 μg/mL gentamicin and streptomycin and 20% fetal calf serum) and then distributed to five 96-well microculture plates (Corning-Costar, Cambridge, MA), which was previously incubated with a feeder layer of peritoneal macrophages. Eleven days after the fusion, cell-free culture supernatants were determined for the presence of anti-tetracycline antibody using a combination of noncompetitive and competitive ELISA. Well cultures resulting in a strong positive response and showing significant anti-mycotoxin reactivity were selected for cloning by limiting dilution using HT-RPMI 1640 medium (supplemented with 10 μg/mL gentamicin and streptomycin and 20% fetal calf serum). Stable antibody producing clones were expanded in RPMI 1640 medium (supplemented with 10 μg/mL gentamicin and streptomycin and 20% fetal calf serum) and cryopreserved in liquid nitrogen. For preparing ascites fluid, mature female BALB/c mice were injected (i.p) with 0.50 mL of paraffin, which was, 7 days later, followed by receiving i.p injection of the hybridoma cells suspended in RPMI 1640 medium. Ascites fluid was collected 10 days after the injection and then stored at –20 °C until use. Purification of monoclonal antibodies was achieved by saturated ammonium sulfate method followed by HiTrap protein A column purification. The isotypes of antibodies were determined by Pierce rapid ELISA mouse antibody isotyping kit (Thermo Scientific, West Palm Beach, FL).

3. Preparation of anti-penicillin receptor

The anti-penicillin receptor PBP-6 was prepared by gene recombination, E. coli expression and purification. Detailed procedures are as follows: The sequence of

penicillin-binding protein 6 was constructed by the method of gene synthesis. Specifically, the primers designed according to the targeted sequence were connected with the restriction sites NdeI and XhoI, which were used for PCR amplification with the synthesized gene as a template. The PCR products were electrophoresed on a 1% agarose gel, and then recovered by a gel recovery kit. The reclaimed PCR products and prokaryotic expression vector pET28a were respectively digested with NdeI and XhoI at 37 °C for 2 h, and then both of them were recovered with a gel recovery kit. The recovered gene products and pET28a were ligated with T4 ligase at 16 °C for 2 h. All ligation products were transformed into DH5α competent cells which were then planted on the inverted LB plate containing kanamycin and cultured at 37 °C overnight. The monoclonal colonies were picked for PCR identification, and the positive ones were used for plasmid extraction and the identification of double enzyme digestion. DNA sequencing was performed for the correct monoclonal colonies identified by double digestion. The correct constructed recombinant plasmid was transformed into E. coli strain BL21 (DE3) which was then cultured inverted at 37 °C overnight. The monoclonal strain was picked for the analysis of protein expression level. And the monoclonal strain with high protein expression identified by SDS-PAGE gel was saved as glycerol bacteria. After expanded cultured and induced for expression, the bacteria were collected and saved. The protease inhibitor was added into the bacterial suspension. Then, the bacteria were collected and supplemented with the protease inhibitor. After ultrasonication, the precipitate of bacteria was removed, and the supernatant was filtered by membranes and purified with a Ni affinity column. The purified target protein was collected and detected for its purity by SDS-PAGE gel. The target protein with purity > 90% was dialyzed and concentrated desalting overnight, and then saved in liquid nitrogen.

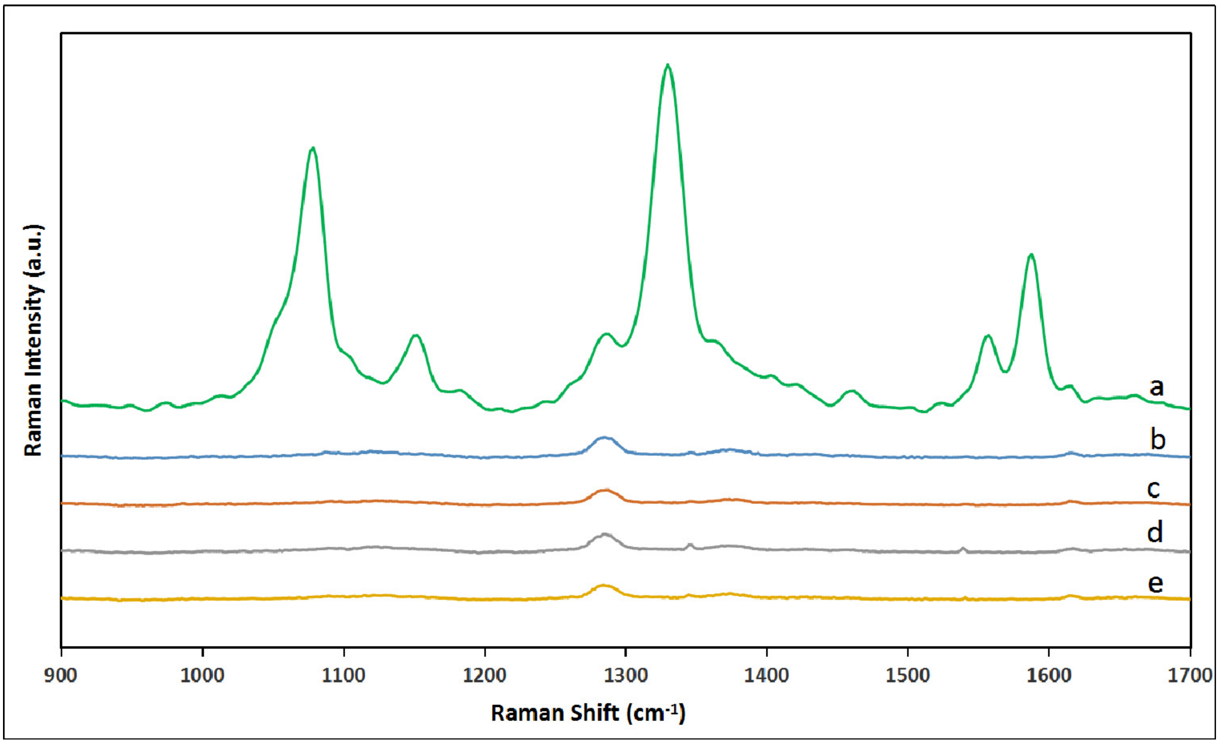

Figure S1. The specificity of the SERS-LFIA for tetracycline and penicillin. The a-e in the figure represent as follow. (a): Tetracycline-BSA and ampicillin-BSA as the capture reagents on test line; (b): BSA as the capture reagent on test line; (c): No anti-tetracycline antibody or anti-β-lactam receptor on SERS nanoprobes as detection reagents; (d): Anti-kanamycin mAb on SERS nanoprobe as detection reagent; (e): Blank test strip.

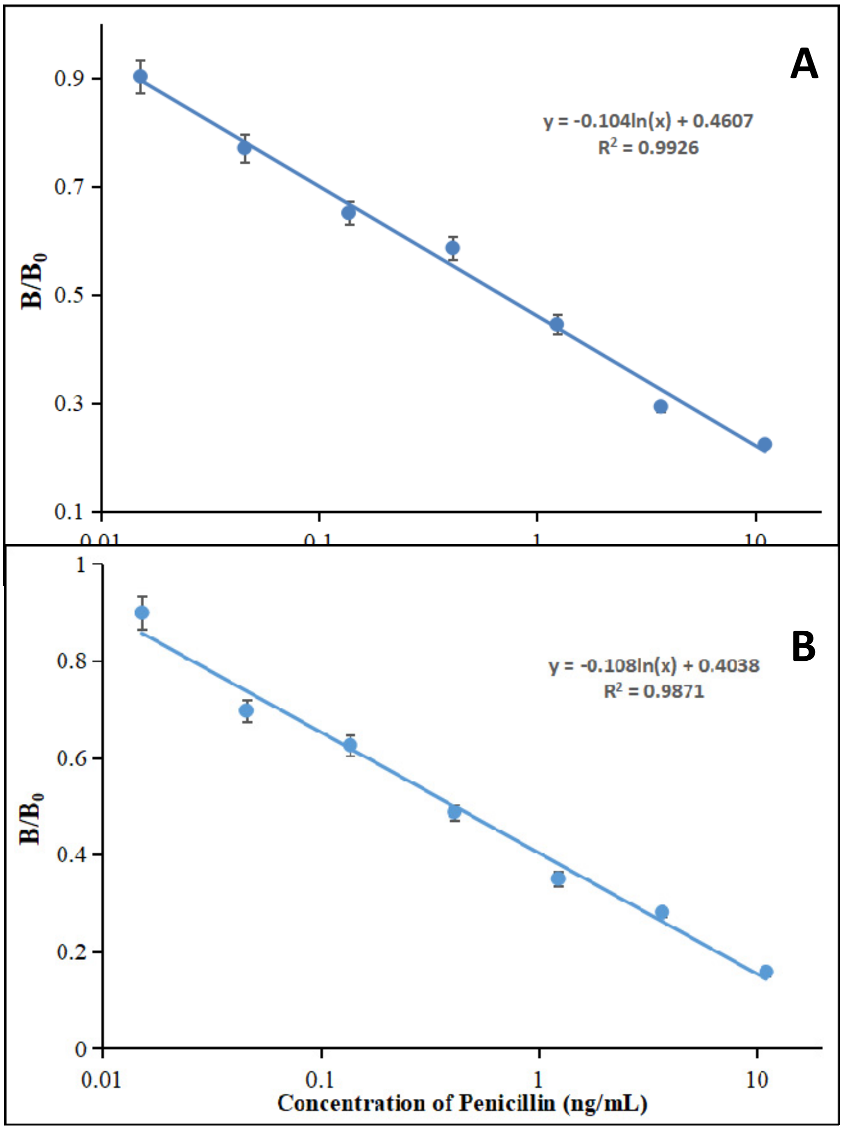

Figure S2. The calibration curves of tetracycline (A) and penicillin (B) in milk by SERS-based lateral flow immunosensor.

Table S1. The IC_{50} and cross-reactivity values of anti-tetracycline mAb and anti- penicillin receptor.

Analyte	IC_{50} values (ng/mL)	Cross-reactivity (100%)
Anti-tetracycline mAb		
Tetracycline	1.62	100
Oxytetracycline	2.79	58
Docycycline	2.52	64
Chlortetracycline	1.42	114
Anti-penicillin receptor		
Penicillin	1.77	100
Ampicillin	2.06	86
Amoxicillin	2.19	81
Oxacillin	2.81	63
Cloxacillin	5.21	34
Nafcillin	5.06	35
Cefquinome	5.53	32
Cephacetrile	7.38	24
Cefalonium	1.42	125
Cefoperazone	2.81	63
Cefepime	5.71	31
Ceftiofur	19.89	9
Cefalexin	12.12	15

Table S2. The reproducibility of SERS-based lateral flow immunosensor for tetracycline and penicillin in milk.

Analyte	Spiked cocentration (ng/mL)	Measured concentration (pg/mL)			CV (%)
		Batch 1 (n=4)	Batch 2 (n=4)	Batch 3 (n=4)	
Tetracycline	0.5	0.41	0.45	0.57	17.5
	5.0	4.7	5.6	6.2	13.3
Penicillin	0.5	0.46	0.52	0.42	10.8
	5.0	4.5	5.9	5.1	13.6

Note: Each batch of test used different SERS nanoprobe and lateral flow strip.

References

1. Bacigalupo, M.A.; Meroni, G.; Secundo, F.; Lelli, R. Time-resolved fluoroimmunoassay for quantitative determination of ampicillin in cow milk samples with different fat contents. *Talanta* **2008**, *77*, 126-130.
2. Broto, M.; Matas, S.; Babington, R.; Marco, M.P.; Galve, R. Immunochemical detection of penicillins by using biohybrid magnetic particles. *Food Control* **2015**, *51*, 381-389.
